# Supplementary material for: Effects of Microbial Transfer during Food-Gut-Feces Circulation on the Health of Bombyx mori
Source: Microbiol Spectr. 2022 Nov 1;10(6):e02357-22. doi: 10.1128/spectrum.02357-22 (PMC9769633; doi:10.1128/spectrum.02357-22)
Supplement: Supplemental file 1 — Supplemental methods and Fig. S1 to S10. Download spectrum.02357-22-s0001.pdf, PDF file, 2.8 MB [file spectrum.02357-22-s0001.pdf]

Supplementary Material

1 Supplementary Materials and Methods

Experimental design

The fresh weight of silkworms was measured at the beginning of each instar stage (n = 3, 100 individuals per sample value to average one silkworm). Instar duration was measured as the time from the beginning of one instar stage to the beginning of the next instar stage. After the pH meter was calibrated, each fresh sample was fully ground and added to 10 times the volume of deionized water, then the pH was detected using a pH meter (LeiciPHS-3E; Shanghai INESA Scientific Instrument Co., Ltd., Shanghai, China). Disease incidence was counted as the proportion of silkworms with the bacterial disease to the total number of silkworms at the beginning of the 5th-instar (100% death if no other treatment was applied). Microbial diversity was analyzed using an amplified region of the 16S rRNA gene (799F–1193R).

Initially, this study compared the phenotypic data and intestinal microbial diversity of healthy silkworms fed different foods, including those fed an artificial diet without antibiotic (Art group, also referred to as the Art_Healthy or Healthy group) and those fed mulberry leaves (Mul group, also referred to as the Mul_Healthy group) (Supplementary Fig. S9a).

Next, this study examined the gut microbial signature of bacterial diseased silkworms. For simplicity and distinction, we set healthy silkworms fed an artificial diet without antibiotics as the Healthy group (also referred to as the Art or Art_Healthy group), diseased silkworms without antibiotics as the Sick group (also referred to as Art_Sick), healthy silkworms with antibiotics (0.01% florfenicol) as the Healthy (+) group, and diseased silkworms with antibiotics as the Sick (+) group. The same diseased silkworms fed a diet ofmulberry leaves were designated as the Mul_Sick group (Supplementary Fig. S9b).

Finally, we performed microbial diversity analysis on the different foods fed to the silkworms in this study. The remaining artificial feed without antibiotics was designated as the Co_Food group (also referred to as the Food group in Fig. 6). This feed was consumed by 5th-instar healthy silkworms for two days, and had traces of the feeding process of silkworm climbing and feces fermentation, simulating the actual production of the breeding factory. For comparison, artificial feeds without antibiotics placed alone under the same environmental conditions for two days were set as the Mono_Food group, with no feces and no silkworm activity, and those with antibiotics were set as the Mono_Food (+) group (Supplementary Fig. S9c). Young mulberry leaves suitable for 1st- to 3rd-instar silkworms were designated as the Leaf_L1L3 group, and old mulberry leaves suitable for 4th- to 5th-instar silkworms were designated as the Leaf_L4L5 group.

To analyze the transfer of bacteria in the Food-Gut-Feces cycle more clearly and concisely, we selected the artificial feed without antibiotics with traces of the feeding process as the Food group (also referred to as Co_Food), the gut of healthy silkworms fed an artificial diet without antibiotics as the Gut group (also referred to as the Art or Healthy group), and the fresh feces of healthy silkworms fed an artificial diet as the Feces Group (Supplementary Fig. S9d)

2 Supplementary Figures

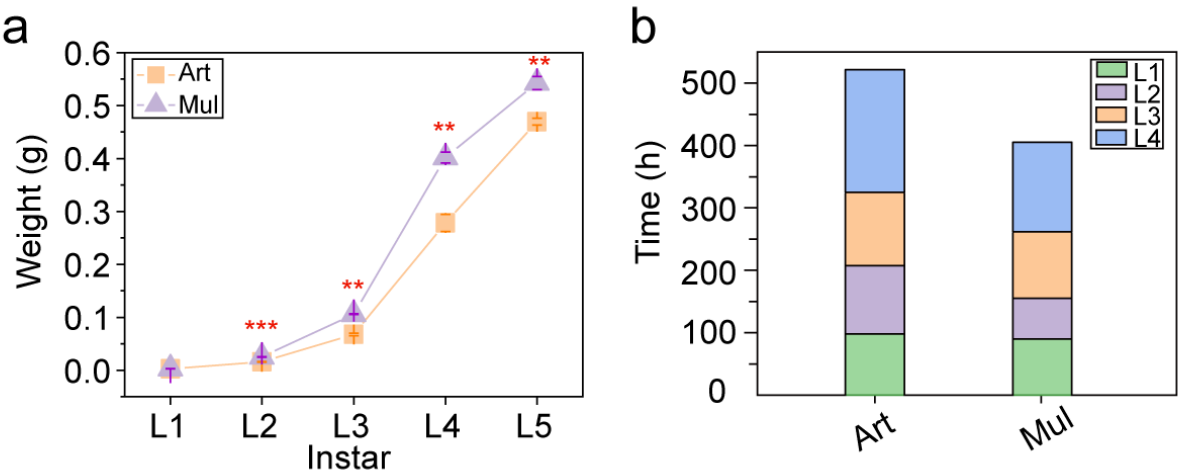

Supplementary Fig. S1 Development of silkworms fed different diets. (**a**) The weight change of silkworms with instar. The asterisk (*) indicates a significant difference $P < 0.05$, (**) indicates $P < 0.01$, and (***) indicates $P < 0.001$. N=3 (100 individuals per sample value to average one silkworm), bars represent Mean ± Standard error, one-way analysis of variance followed by Bonferroni's post-hoc test). (**b**) Stacked graph of instar duration. Stacked blocks of the same color correspond to the same instar. The larger the accumulation area, the longer the duration for the instar. Silkworms fed an artificial diet (Art group), and mulberry leaves (Sick group).

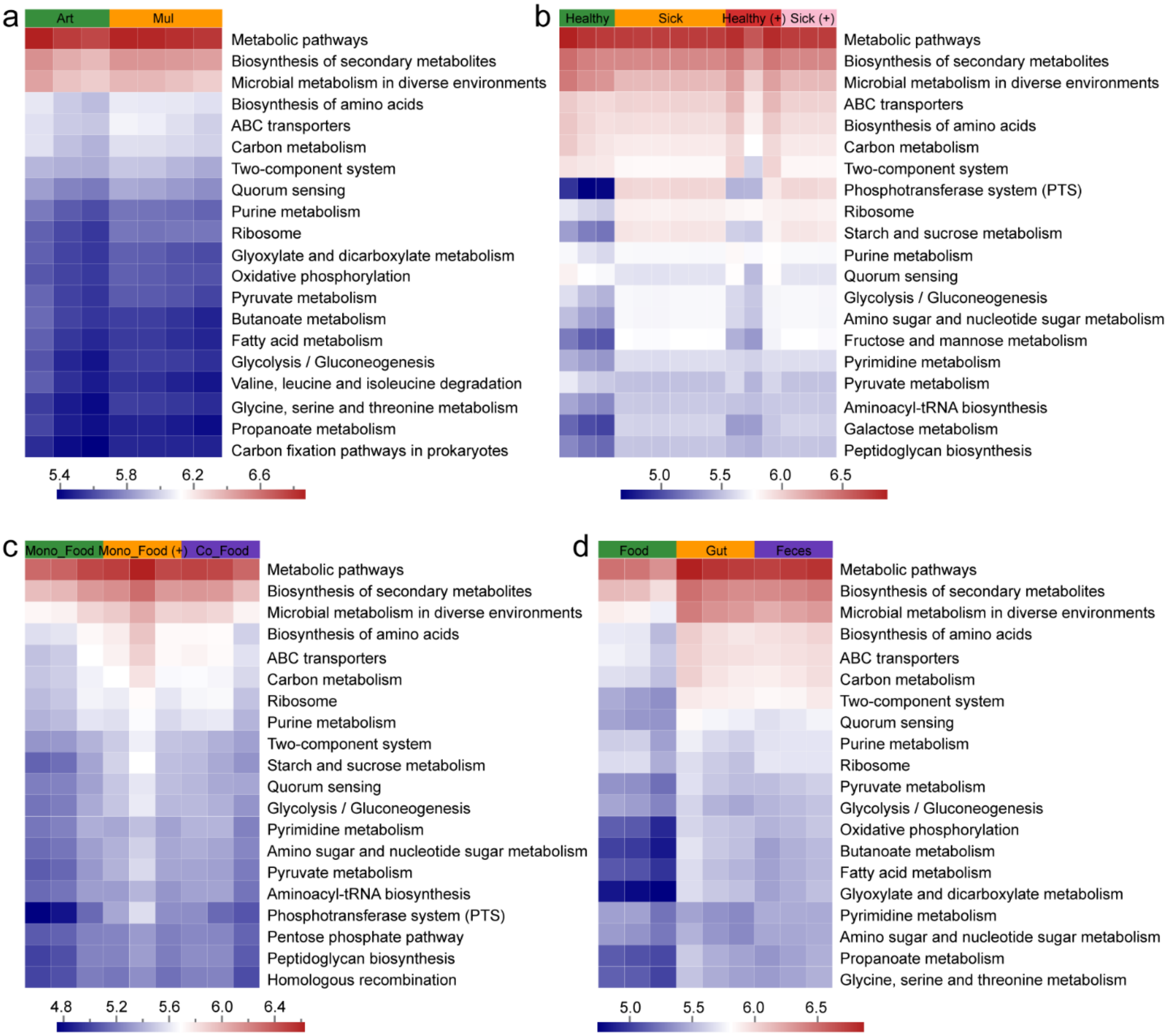

Supplementary Fig. S2 Heatmap of KEGG pathway level 3 by PICRUSt2 function prediction. (**a**) Comparison of silkworm fed with mulberry leaves and artificial feed, (**b**) healthy and diseased silkworms, (**c**) different foods, and (**d**) the Food–Gut–Feces.

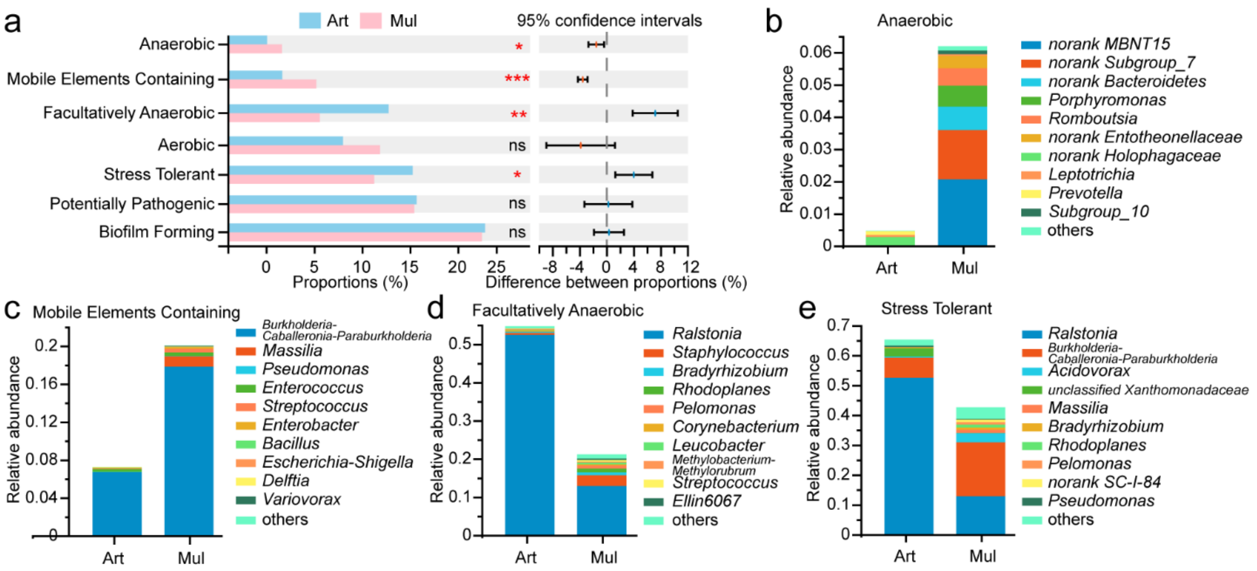

Supplementary Fig. S3 BugBase phenotype prediction of silkworms fed different diets. (a) The proportion of different phenotypes in each group. The asterisk (*) indicates a significant difference $P < 0.05$, (**) indicates $P < 0.01$, and (***) indicates $P < 0.001$. (N=3, bars represent Mean ± Standard error, analysis by student's t test). **(b)**–**(e)** Species-phenotype contribution plot of phenotypes with significantly differences in (a).

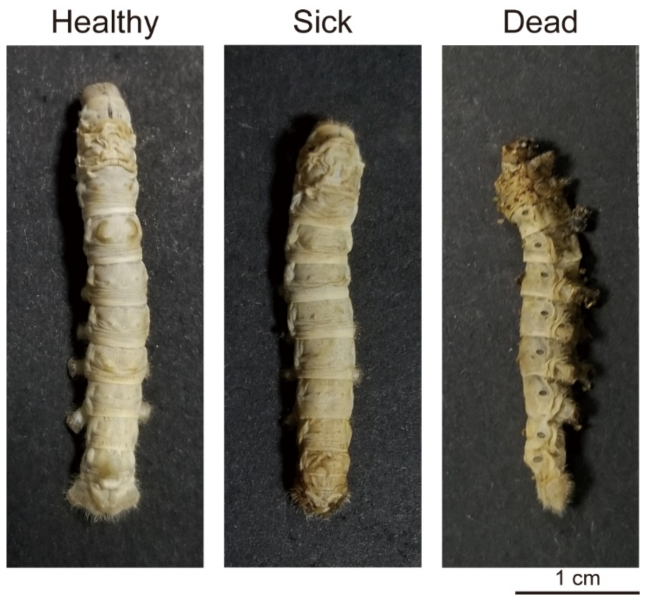

Supplementary Fig. S4 Bacterial disease phenotype of silkworms fed an artificial diet. Depicts the individual phenotype of the 5th-instar silkworm given artificial feed. Scale bar represents 1 cm. At first, the body turned slightly yellow; then the tail decayed, and the body shortened. At the time of death, the abdomen swelled slightly and the whole body was limp.

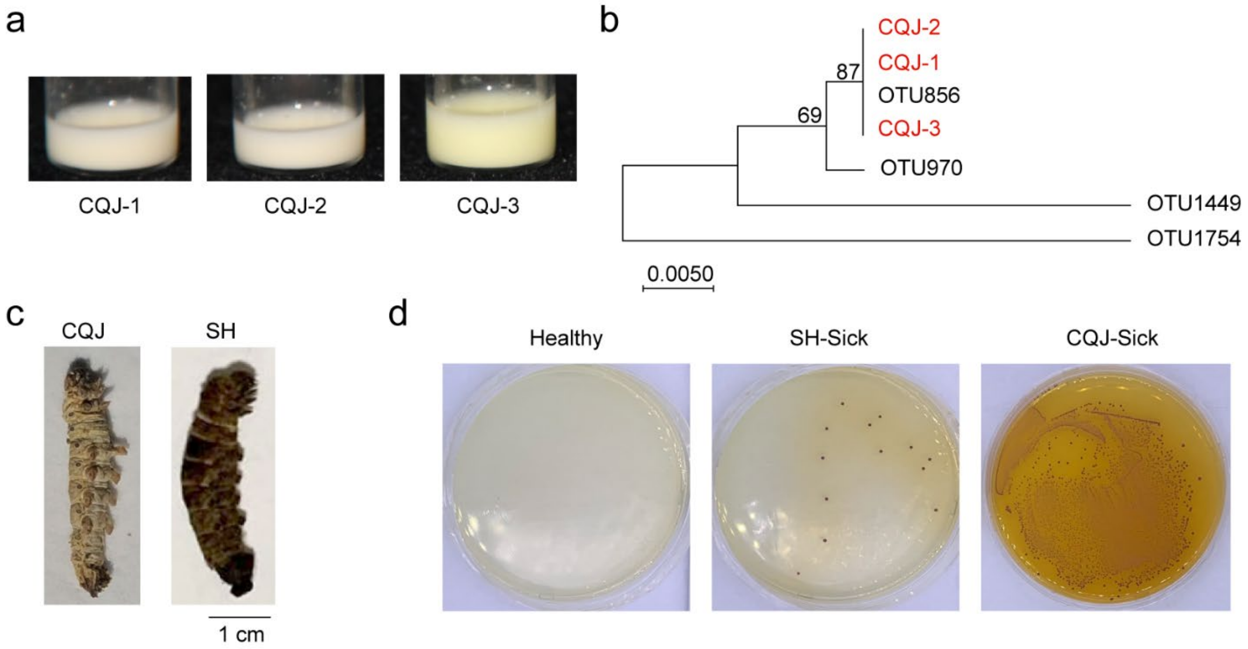

Supplementary Fig. S5 Isolation, inoculation and validation of *Enterococcus*. (**a**) Phenotypic of bacterial cell enrichment of three different strain of *Enterococcus. mundtii* (CQJ-1, CQJ-2, and CQJ-3). (**b**) Molecular evolutionary tree for three *E. mundtii* strains and 4 OTUs of *Enterococcus* genus. The tree was built using the same region gene as microbial diversity (799F and 1193R) by the neighbor joining method with a bootstrap value of 1000. (**c**) Phenotype of diseased silkworms inoculated with bacteria. 5th-instar silkworm fed an artificial diet after inoculation. CQJ, inoculated *E. mundtii* CQJ-1, CQJ-2, and CQJ-3 (they had the same phenotype); SH, inoculated *Staphylococcus. sciuri* SH. (**d**) The number of *Enterococcus* growth on *Enterococcus* chromogenic medium at 37 °C for 48h of dark inoculated 50 μL intestinal fluid diluent (serial dilution 10^3 times). Healthy, *Enterococcus* in gut of the healthy silkworms fed an artificial diet without inoculation; SH-Sick, *Enterococcus* in gut of the diseased silkworms fed an artificial diet with inoculation *S. sciuri* SH. CQJ-Sick, *Enterococcus* in gut of diseased silkworm fed an artificial diet with inoculation *E. mundtii* CQJ-1, CQJ-2, and CQJ-3 (they had the same result).

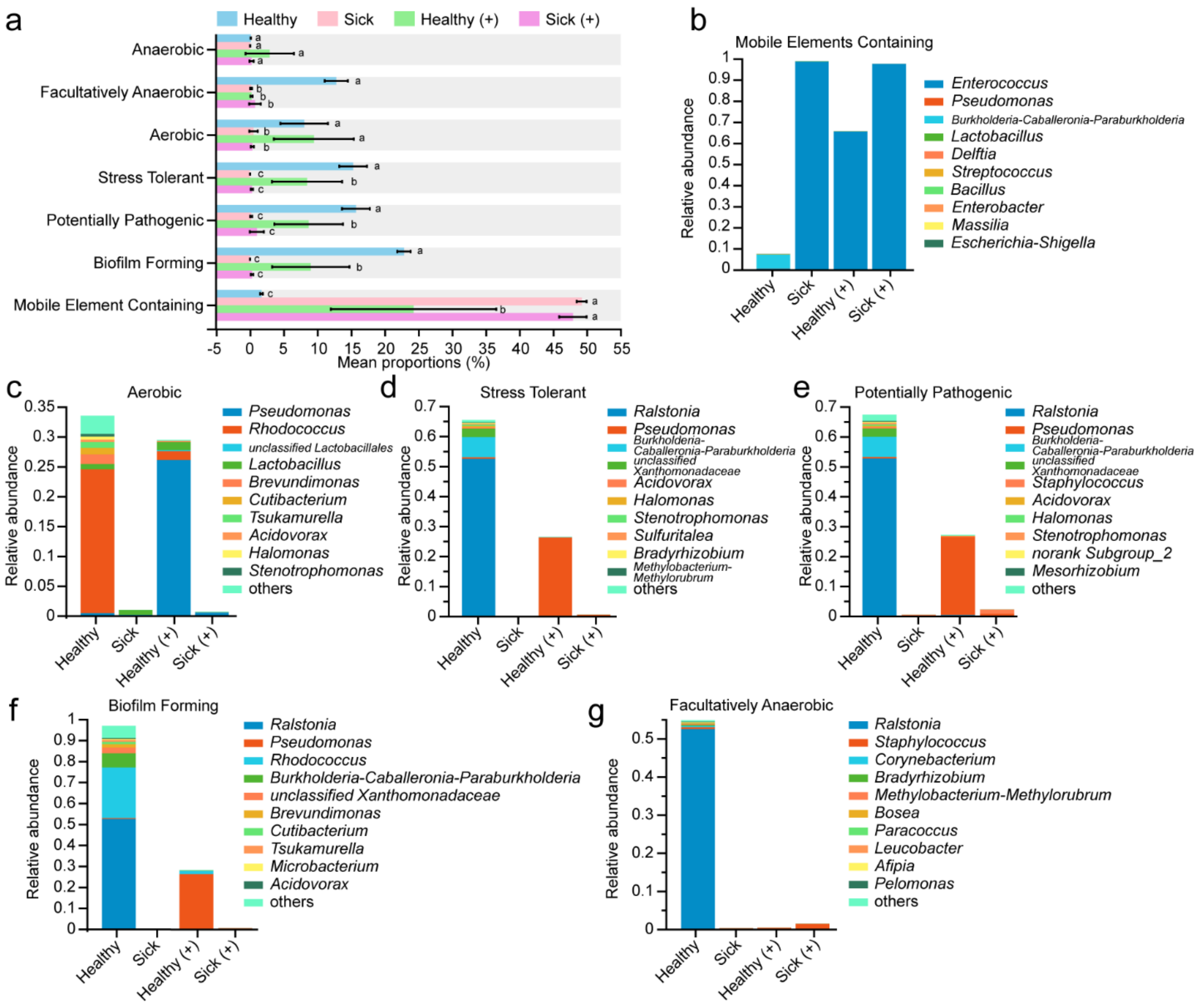

Supplementary Fig. S6 BugBase phenotype prediction of the healthy and disease groups fed an artificial diet with or without antibiotics. (**a**) The proportion of different phenotypes in each group. Different letters (a, b, and c) above the bars indicate a significant difference at $P < 0.05$ (N=3, bars represent Mean ± Standard error, analysis by student's *t* test). (**b**)–(**g**) Species-phenotype contribution plot of phenotypes with significantly differences in (a).

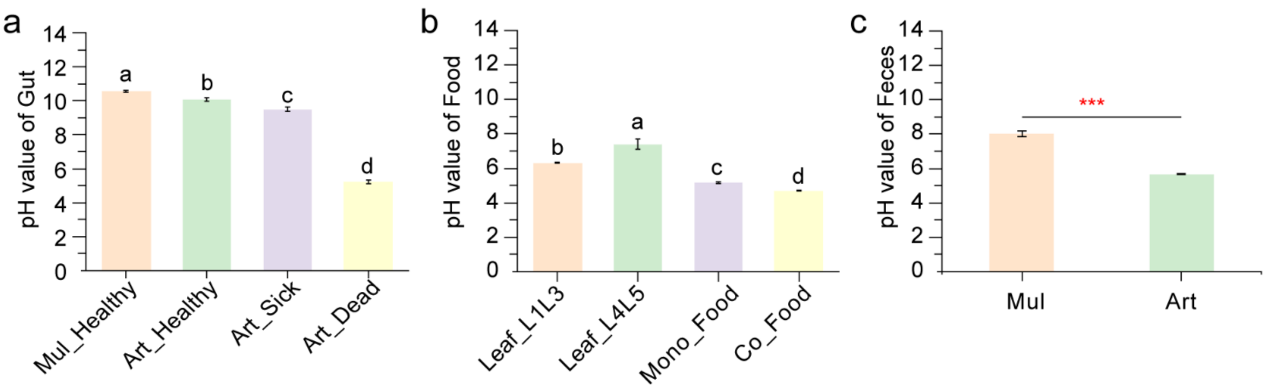

Supplementary Fig. S7 pH Changes in Gut (a), Food (b), and Feces (c). After the pH meter was calibrated, each fresh sample was fully ground and added to 10 times the volume of deionized water, then the pH was detected using a pH meter. Different letters (a, b, and c) above the bars indicate a significant difference at $P < 0.05$ and the asterisk (***) indicates $P < 0.001$. N=3 (100 individuals per sample value to average one silkworm), bars represent Mean ± Standard error, one-way analysis of variance followed by Bonferroni's post-hoc test. Leaf_L1L3, young mulberry leaves suited for 1st- to 3rd-instar silkworms; Leaf_L4L5, old mulberry leaves suited for 4th- to 5th-instar silkworms.

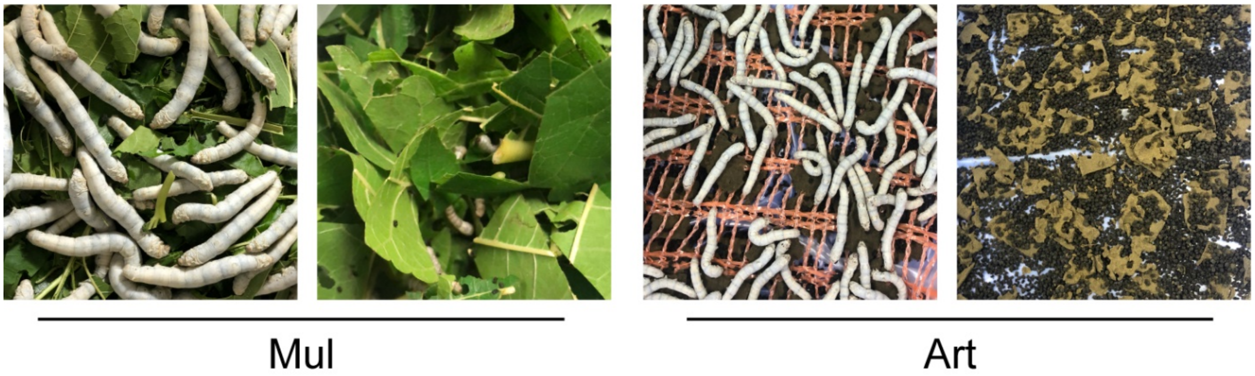

Supplementary Fig. S8 Environment of silkworm growth by different foods. Mul, represents feed mulberry leaves, and Art, feed artificial diet.

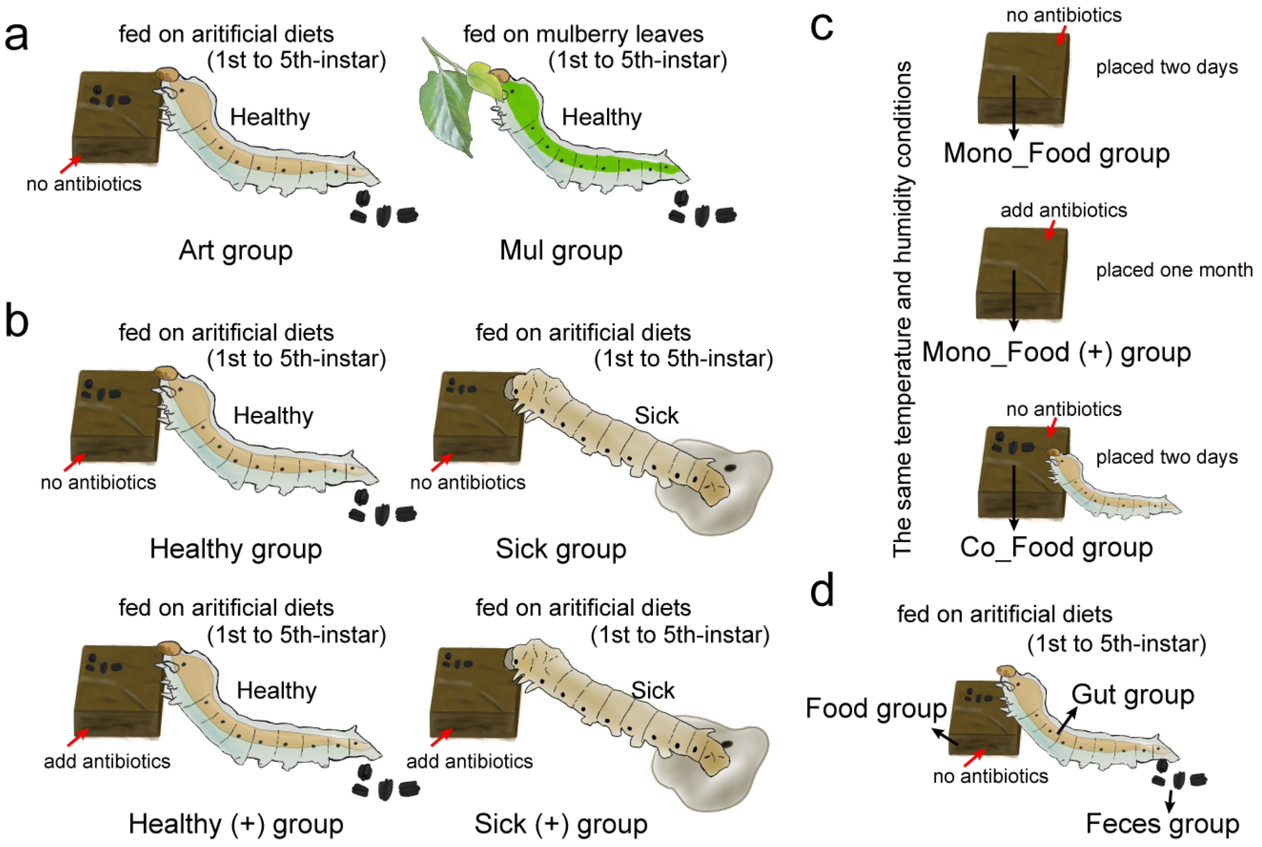

Supplementary Fig. S9 Experimental design. (**a**) Comparison of silkworm fed with mulberry leaves and artificial feed, (**b**) healthy and diseased silkworms, (**c**) different foods, and (**d**) the relationship among Food–Gut–Feces.

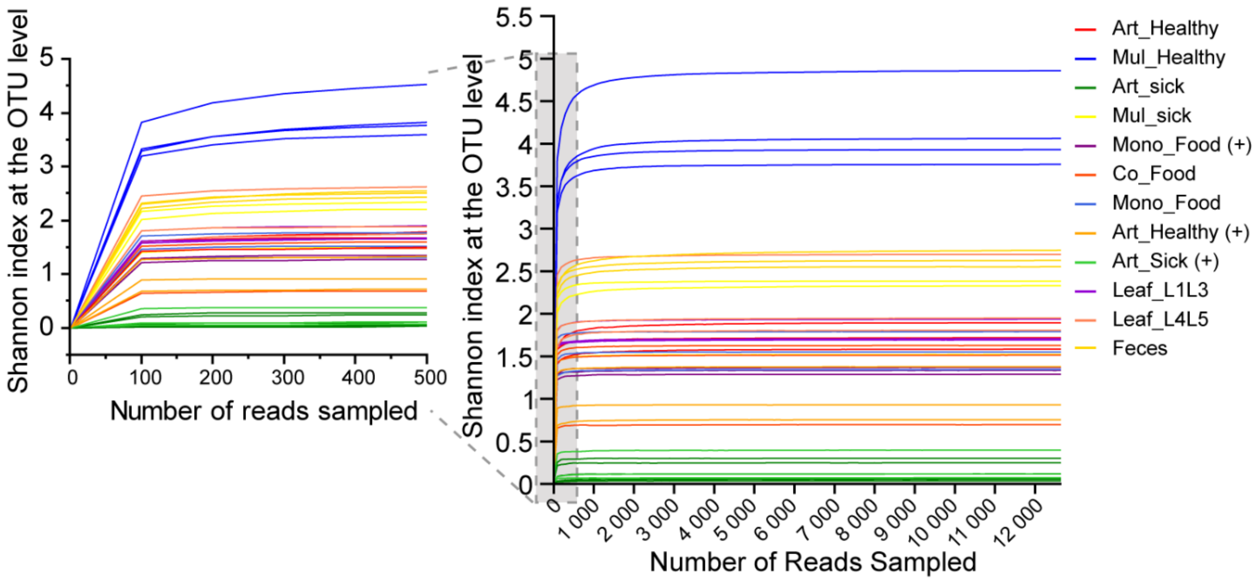

Supplementary Fig. S10 Microbiota rarefaction curves. Shannon index plot at the operational taxonomic unit (OTU) level with 97% similarity. An enlarged view of the X-axis on the left.

2 Tables

Table S1: **Alpha diversity indexes** for (**a**) the gut groups fed different diets, (**b**) the healthy and disease groups fed the artificial diets with or without antibiotics, and (**c**) the food groups under different storage conditions. Different letters (a, b, and c) above the bars in the same index indicate a significant difference at $P < 0.05$ (N=3, bars represent Mean ± Standard error, analysis by student's *t* test). **See excel file named Supplementary file Table S1.**

Table S2: *Enterococcus* distribution in each sample. See excel file named Supplementary file Table S2.

Table S3: **Distribution of bacteria in each sample**. **See excel file named Supplementary file Table S3.**

Table S4: **KO functional abundance in each sample. See excel file named Supplementary file Table S4.**
